# Monitoring the tissue perfusion during hemorrhagic shock and resuscitation: tissue-to-arterial carbon dioxide partial pressure gradient in a pig model

**DOI:** 10.1186/s12967-021-03060-5

**Published:** 2021-11-14

**Authors:** Yusuke Endo, Taku Hirokawa, Taku Miyasho, Ryosuke Takegawa, Koichiro Shinozaki, Daniel M. Rolston, Lance B. Becker, Kei Hayashida

**Affiliations:** 1 The Feinstein Institutes for Medical Research, Northwell Health System, 350 Community Drive, Manhasset, NY 11030 USA; 2 School of Veterinary Medicine, Rakuno Gakuen University, Hokkaido, Japan; 3 Department of Emergency Medicine, North Shore University Hospital, Northwell Health, Manhasset, NY USA; 4 Zucker School of Medicine at Hofstra/Northwell, New York, NY USA

**Keywords:** Hemorrhage, Hemorrhagic shock, Resuscitation, Transcutaneous partial pressure monitoring of carbon dioxide partial pressure, Transcutaneous partial pressure monitoring of oxygen, Catheterization

## Abstract

**Background:**

Despite much evidence supporting the monitoring of the divergence of transcutaneous partial pressure of carbon dioxide (tcPCO_2_) from arterial partial pressure carbon dioxide (artPCO_2_) as an indicator of the shock status, data are limited on the relationships of the gradient between tcPCO_2_ and artPCO_2_ (tc-artPCO_2_) with the systemic oxygen metabolism and hemodynamic parameters. Our study aimed to test the hypothesis that tc-artPCO_2_ can detect inadequate tissue perfusion during hemorrhagic shock and resuscitation.

**Methods:**

This prospective animal study was performed using female pigs at a university-based experimental laboratory. Progressive massive hemorrhagic shock was induced in mechanically ventilated pigs by stepwise blood withdrawal. All animals were then resuscitated by transfusing the stored blood in stages. A transcutaneous monitor was attached to their ears to measure tcPCO_2_. A pulmonary artery catheter (PAC) and pulse index continuous cardiac output (PiCCO) were used to monitor cardiac output (CO) and several hemodynamic parameters. The relationships of tc-artPCO_2_ with the study parameters and systemic oxygen delivery (DO_2_) were analyzed.

**Results:**

Hemorrhage and blood transfusion precisely impacted hemodynamic and laboratory data as expected. The tc-artPCO_2_ level markedly increased as CO decreased. There were significant correlations of tc-artPCO_2_ with DO_2_ and COs (DO_2_: r = − 0.83, CO by PAC: r = − 0.79; CO by PiCCO: r = − 0.74; all P < 0.0001). The critical level of oxygen delivery (DO_2crit_) was 11.72 mL/kg/min according to transcutaneous partial pressure of oxygen (threshold of 30 mmHg). Receiver operating characteristic curve analyses revealed that the value of tc-artPCO_2_ for discrimination of DO_2crit_ was highest with an area under the curve (AUC) of 0.94, followed by shock index (AUC = 0.78; P < 0.04 vs tc-artPCO_2_), and lactate (AUC = 0.65; P < 0.001 vs tc-artPCO_2_).

**Conclusions:**

Our observations suggest the less-invasive tc-artPCO_2_ monitoring can sensitively detect inadequate systemic oxygen supply during hemorrhagic shock. Further evaluations are required in different forms of shock in other large animal models and in humans to assess its usefulness, safety, and ability to predict outcomes in critical illnesses.

## Background

Hemorrhagic shock is a life-threatening condition, with a significant loss of intravascular volume, decreased cardiac output (CO), decreased tissue perfusion pressure, and cellular hypoxia, resulting in multiple organ damage and death [1, 2]. The ultimate goal of resuscitation for hemorrhagic shock is the rapid control of the source and the restoration of effective tissue perfusion, oxygenation, and cellular metabolism [2]. Thus, prompt assessment of the adequacy of tissue perfusion is essential for the early identification and intervention of hemorrhagic shock.

The pulmonary artery catheter (PAC) provides continuous monitoring of comprehensive hemodynamic parameters, including stroke volume, CO, mixed venous oxygen saturation, and intracardiac pressure, with several additional calculated variables to guide diagnosis and treatment [3]. The pulse index continuous cardiac output (PiCCO) is another efficient and advanced hemodynamic monitoring system that integrates various hemodynamic variables through intra-arterial and central venous catheterization [4]. However, these techniques are invasive, with no clear evidence of improved outcomes associated with their use to guide therapy [5, 6].

Monitoring of transcutaneous partial pressure of oxygen (tcPO_2_) and carbon dioxide (tcPCO_2_), which can non-invasively assess arterial partial pressure of oxygen (artPO_2_) and carbon dioxide (artPCO_2_), respectively, has been investigated for several decades [7–15]. However, in clinical practice, adherence to their use remains low [7]. Both measures are indicators of tissue perfusion adequacy during shock [7, 12, 16, 17]. During low-flow shock, tcPO_2_ can detect tissue hypoxia or inadequate perfusion [18, 19]. In particular, tcPCO_2_ and artPCO_2_ mismatch and decoupling occurs during shock states [20], and increased tcPCO_2_ is associated with poor outcomes in critically ill patients [12, 21]. These observations suggest that the gradient between tcPCO_2_ and artPCO_2_ (tc-artPCO_2_) measured in a minimally invasive manner (i.e., with only arterial puncture needed), is useful as a rapid and accurate measurement during shock.

Despite much evidence supporting the monitoring of the divergence of tcPCO_2_ from artPCO_2_ as an indicator of the shock status, data are limited on the systemic oxygen metabolism and hemodynamic parameters, which can be obtained by PAC or PiCOO in shock states. In this study, we tested the hypotheses that (1) significant associations between tc-artPCO_2_ and various hemodynamic parameters of PAC or PiCCO exist and (2) tc-artPCO_2_ could be an early and sensitive method to detect inadequate tissue perfusion in a pig model of progressive hemorrhage shock and resuscitation.

## Methods

The experimental procedures were approved by the ethics committee for animal experiments at Rakuno Gakuen University (Protocol Number: VH19B14). Care and handling of the animals were performed in accordance with the guidelines of the National Institutes of Health.

### Animal preparation

All studies were performed by a qualified and experienced research team using female pigs (Landrace × Large White × Duroc [LWD], weighing 29–34 kg). Food was withheld from the animals for 12 h before the start of the experiment; however, water was allowed 6 h prior. After getting used to the environment and following physical examination by a veterinarian, the animals were sedated by an intramuscular injection of medetomidine hydrochloride (40.0 μg/kg), midazolam (0.2 mg/kg), and butorphanol tartrate (0.2 mg/kg). Tracheal intubation was performed after induction of anesthesia using propofol, and general anesthesia was maintained with inhaled 2.0% sevoflurane (Sevoflo®, Dainippon-Sumitomo Pharma, Osaka, Japan). Anesthetics were adapted if the depth of anesthesia was insufficient. Following anesthesia induction, the pigs were placed in a supine position and administered a fluid infusion of lactated Ringer’s solution (LR, Terumo Co., Tokyo, Japan) at 10 mL/kg/h, through a 22-gauge catheter (Supercath, Medikit Co., Tokyo, Japan) placed in the right marginal ear vein. All animals were mechanically ventilated in volume-control mode (Flow-i, Maquet, Sonia, Sweden) after intravenous administration of 2 mg/kg vecuronium (Musculate®, Fuji Pharma Co., Tokyo, Japan), followed by a constant-rate infusion at 0.1 mg/kg/h, administered through the left marginal ear vein. The ventilation settings were 8–10 mL/kg tidal volume with a respirator, with positive end-expiratory pressure set at 5 cm H_2_O. The fraction of inspired oxygen (F_I_O_2_) was set at 0.5, with an inspiration to expiration ratio of 1:2; the respiratory rate was adjusted to 16–20/min to maintain artPCO_2_ 50 ± 5 mmHg. Body temperature was maintained at 37.0 °C ± 0.5 °C using a heating pad.

### Experimental protocol

**Fig. 1:**
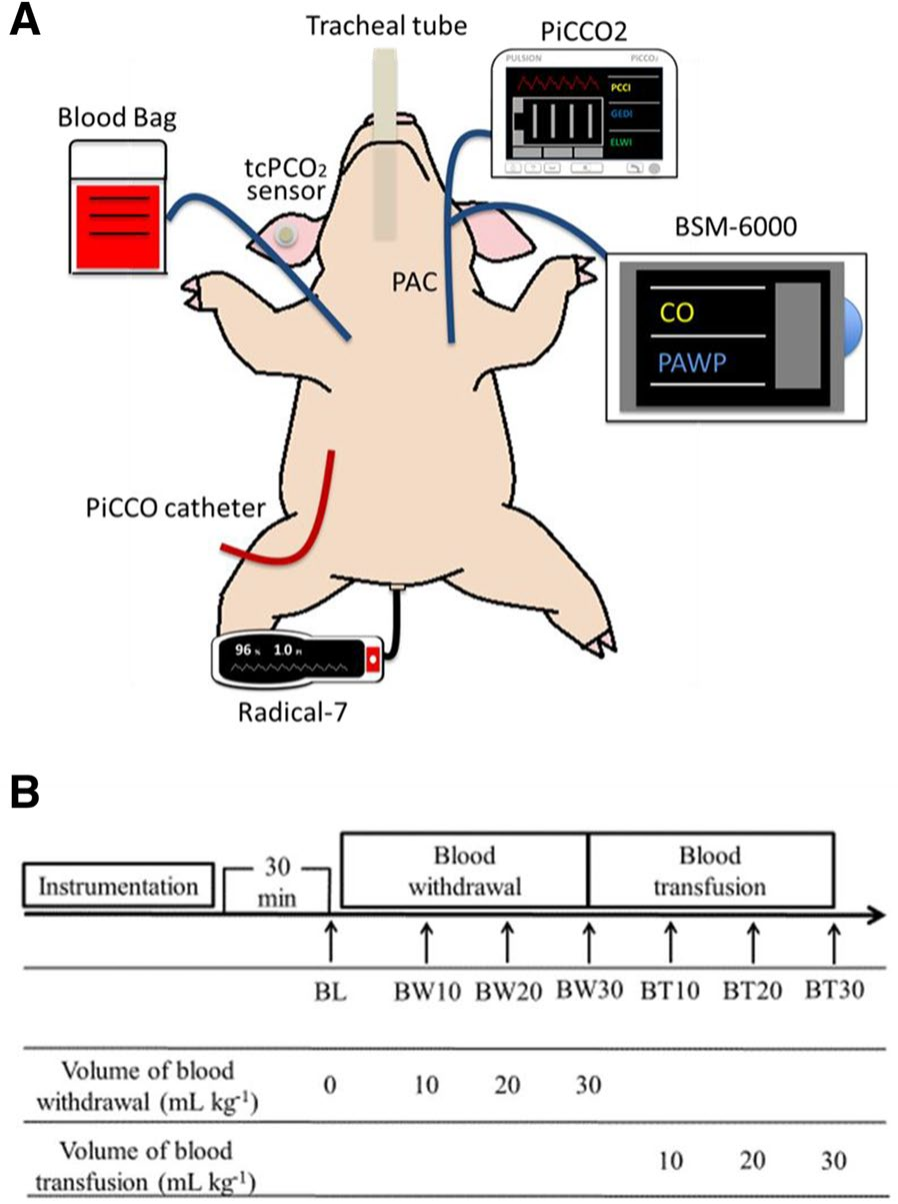
Experimental protocol. **A** A schema of the pig hemorrhagic shock and resuscitation model. The animal was intubated and mechanically ventilated. A transcutaneous carbon dioxide monitor was attached on the right ear. A pulmonary artery catheter was inserted into the left internal jugular vein. The right femoral artery was catheterized with a thermistor-tipped PiCCO catheter. Arterial blood pressure was measured via the femoral artery using a PiCCO catheter. A double-lumen central venous catheter was inserted into the right jugular vein and positioned at the cranial end of the superior vena cava for blood withdrawal and transfusion. A transcutaneous, multi-wavelength pulse oximetry monitor was attached to the tail after clipping the hair. **B** Experimental protocol. tcPCO_2_, transcutaneous partial pressure of carbon dioxide; PAC, pulmonary artery catheter; PiCCO, pulse index continuous cardiac output; BL, baseline; BW, blood withdrawal; BT, blood transfusion

After each catheterization was completed, all animals were allowed to stabilize for 30 min, and each initial value was regarded as the baseline value. To induce a progressive hemorrhagic shock status, blood was withdrawn from the central venous catheter and stored in sterile bags with a volume of citrate–phosphate-dextrose-adenine (CPDA-1) appropriate for 30 mL/kg of blood (14 mL CPDA-1 per 100 mL of blood). Blood withdrawal (BW) was performed three times by each 10 mL/kg, and a total of 30 mL/kg was collected. The measurement points where the loss of circulating blood volume reached 10, 20, and 30 mL/kg were defined as BW10, BW20, and BW30, respectively. Next, the animals were resuscitated by transfusing them with the stored blood. Blood transfusion (BT) was performed at intervals of 10 mL/kg, transfusing 30 mL/kg in total. The points where the gain of circulation blood volume reached 10, 20, and 30 mL/kg were defined as BT10, BT20, and BT 30, respectively (Fig. 1). Before each measurement, the animals were allowed to stabilize for 10 min.

### Instrumentations

A 7-Fr PAC (Edward Lifesciences, Irvine, CA, USA) was inserted into the left internal jugular vein and advanced into the pulmonary artery. Assessment of pulmonary artery pressure and wedge waveforms confirmed the correct position of the PAC. Simultaneously, the right femoral artery was catheterized with a thermistor-tipped 4-Fr PiCCO catheter (PV2014L16, Pulsion Medical Systems AG, Munich, Germany) connected to the PulsioFlex platform (Pulsion Medical System, Munich, Germany). Arterial blood pressure was measured via the femoral artery using a PiCCO catheter. A 6-Fr double-lumen central venous catheter (UK catheter kit UB-0610-W, 21G, 10 cm, Unitika Medical, Osaka, Japan) was inserted into the right jugular vein and positioned at the cranial end of the superior vena cava for blood withdrawal and transfusion. The distal ports of the PAC were connected to the PulsioFlex monitor sensor for the same time as for the measurement of CO via an injection of ice-cold physiological saline 0.9% (Isotonic Sodium Chloride Solution®). All transducers were zeroed and positioned at the level of the right atrium.

### Transcutaneous CO_2_ and O_2_ measurements

The transcutaneous monitoring system (TCM4; Radiometer, Copenhagen, Denmark) provides non-invasive and continuous measurement of tcPO_2_ and tcPCO_2_. Prior to the fixation of transcutaneous sensor (tc Sensor 84; Radiometer, Copenhagen, Denmark), the skin was cleaned, and an adhesive ring with two drops of contact gel was applied, according to the manufacturer’s instructions. Measurements were performed with the animals in the supine position with a skin probe positioned on the right ear. Calibration was conducted automatically, with the temperature of the skin probe set at 44 °C. The tcPCO_2_ and tcPO_2_ values were obtained and recorded before CO measurement, during each hemodynamic stage, by an investigator blinded to the experiments.

### Measurements of hemodynamic parameters

CO was measured by pulmonary artery thermodilution (CO_PATD_: using PAC) and transpulmonary thermodilution (CO_TPTD_: using PulsioFlex); 10 mL of ice-cold physiological saline 0.9% (Isotonic Sodium Chloride Solution®) was used as an indicator and was injected through the left distal port of PAC. PATD and TPTD were measured simultaneously at each time point by the same operator (YE), who was blinded to the transcutaneous CO_2_ and O_2_ measurements and laboratory data. To unify the CO_TPTD_ measurement method and eliminate the nominative effects, CO_TPTD_ was measured by the following methods: (1) each measurement was triplicated, and the averaged values of measurements were used for the analyses, (2) the indicator was injected by the same operator (YE) throughout the overall study period, (3) ΔT in TPTD (the change in blood temperature after indicator injection) was recorded; optimal = ΔT > 0.3, good = ΔT > 0.2, and bad = ΔT < 0.2, to verify the reliability of the measurement method, and (4) the indicator was injected exactly for 2–3 s (to avoid faster or slower injection); and (5) the temperature of the indicator was accurately controlled at 0 °C using an ice and cooler box. In this study, ΔT > 0.3 occurred 38 times, ΔT > 0.2 occurred 4 times, and ΔT < 0.2 did not occur at all, indicating that TPTD at all measurement points was quite accurate according to the TPTD instruction.

Hemodynamic parameters measured by PAC (pulmonary artery thermodilution [PATD]) included the central venous pressure (CVP), pulmonary artery wedge pressure (PAWP), stroke volume (SV_PATD_), and CO (CO_PATD_). Transpulmonary thermodilution [TPTD] parameters obtained by PiCCO technology (Pulsion Medical System, Munich, Germany) included the mean arterial pressure (MAP), SV (SV_TPTD_), CO (CO_TPTD_), global end-diastolic blood volume (GEDV), stroke volume variation (SVV), pulse pressure variation (PPV), cardiac function index (CFI, CO/GEDV), cardiac power output (CPO, mean arterial pressure [mmHg] × CO [L/min] × *Κ*, where Κ = 0.0022 [a conversion factor]). The shock index (SI), defined as heart rate divided by systolic blood pressure, at each measurement point was calculated. SI of > 1.0 was indicative of worsening hemodynamic status and shock [22]. PATD and TPTD could be measured at each time point. SVV was not observed at two time points in one pig for an unknown reason; all other hemodynamic variables displayed each value.

Additionally, a perfusion index (PI), which was derived from pulse oximetry readings (Masimo SET Radical-7TM, Masimo Inc, Irvine, CA, USA), was monitored simultaneously. The Masimo Radical-7 uses transcutaneous, multi-wavelength analysis for non-invasive measurement of arterial oxygen saturation, PI, and pleth variability index (PVI), which are measures of local blood flow. A sensor (LNOP Neo-L, Masimo Inc., Irvine, CA, USA) was attached to the tail after clipping the hair. Esophageal temperature and heart rate were also recorded (BSM-6000; Nihon Kohden Inc., Tokyo, Japan).

### Arterial blood gas analysis

Arterial blood samples were withdrawn anaerobically from the PiCCO catheter and collected in a plastic syringe heparinized with 1000 U/mL of sodium heparin (Novo-heparin for injection, Mochida Pharmaceutical Co., Tokyo, Japan), using an evacuation technique to minimize sample dilution. The blood samples were analyzed by an investigator blinded to the experiments immediately after collection (< 5 min) to measure artPO_2_, artPCO_2_, hemoglobin (Hb), and lactate, using a blood gas analyzer (ABL-90 FLEX; Radiometer, Copenhagen, Denmark).

### Systemic oxygen delivery

An inappropriate level of systemic oxygen delivery (DO_2_) capacity fails to satisfy the metabolic oxygen need in the tissue. It is well established that tissues exhibit a level of systemic DO_2_, known as the critical DO_2_ (DO_2 crit_), below which the extraction cannot increase sufficiently to sustain O_2_ uptake; O_2_ consumption then becomes supply-dependent [23–26]. DO_2_ was calculated using the following equation: DO_2_ (mL/kg/min) = CO × hemoglobin [Hb] × 1.36 × arterial oxygen saturation (SaO_2_) + (partial pressure of oxygen [PaO_2_] × 0.0031) [27]. In this study, tcPO_2_ was used to define DO_2crit_ since tcPO_2_ reflects tissue oxygenation under experimental conditions. Given that the lowest baseline tcPO_2_ associated with survival was 28 mmHg [28], the lowest threshold for tcPO_2_ was set as 30 mmHg. This is consistent with previous studies, which demonstrated that tcPO_2_ values < 25 mmHg reflects a 50% decrease in oxygen consumption, preceding cardiac arrest [29], and that a tcPO_2_ value of 40 mmHg is a critical artPO_2_ in the subcutaneous tissue [30, 31].

### Statistical analysis

Values are expressed as mean ± standard error of the mean. An unpaired two-tailed Student’s t-test or Mann–Whitney U test was performed to compare two independent groups, as appropriate. Linear regression and Bland–Altman analyses were performed to determine the correlation between tcPCO_2_ and artPCO_2_. Repeated one-way analysis of variance (ANOVA) followed by Dunnett's correction and Friedman test followed by Dunn's correction were performed for post-hoc comparisons of normally and non-normally distributed data, respectively. Spearman's correlation coefficients (r) were calculated to evaluate the correlation between any two parameters. To examine the accuracy of tc-artPCO_2_, SI, and arterial lactate for predicting DO_2crit_, receiver operating characteristic (ROC) curve analyses were performed. The area under the curve (AUC) between two pairs of potential predictors was compared using a nonparametric test. Statistical significance was defined as a two-sided p-value of < 0.05. All statistical analyses were performed using GraphPad Prism version 8.3.0 (GraphPad Software, San Diego, CA, USA) and Microsoft Excel (Microsoft 365, Microsoft Corporation, Redmond, WA, USA).

## Results

All six pigs were included, and 42 paired measurements of each of their variables were analyzed. No death occurred during the experiments.

### Hemodynamics and blood gas analysis

**Fig. 2:**
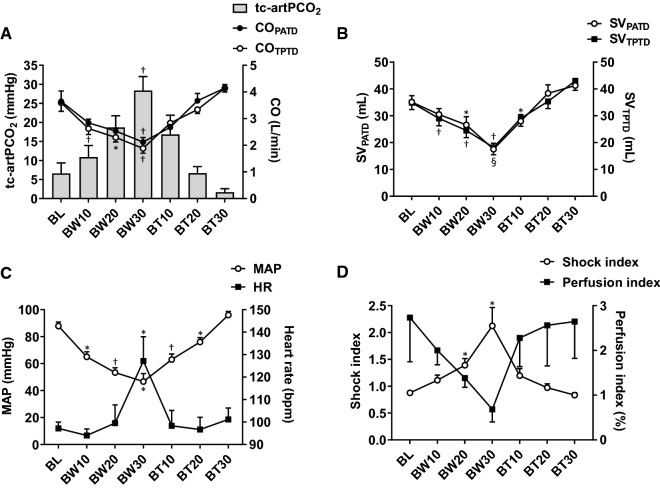
Changes in hemodynamic parameters during hemorrhage shock and resuscitation. **A** Blood withdrawal induced the increments in transcutaneous partial pressure monitoring of oxygen and carbon dioxide (Friedman test, P = 0.0002), and the decrements in cardiac outputs measured by pulmonary artery thermodilution (Friedman test, P < 0.0001) and by transpulmonary thermodilution (Repeated-Measure [RM] ANOVA, F = 31.2, P = 0.0006). These alterations were recovered by blood transfusions. **B** Blood withdrawal induced the decrements in stroke volumes measured by pulmonary artery thermodilution (RM ANOVA, F = 18.41, P < 0.0001) and by transpulmonary thermodilution (RM ANOVA, F = 52.14, P < 0.0001). These alterations were recovered by blood transfusions. **C** Blood withdrawal induced the increments in heart rate (RM ANOVA, F = 4.24, P = 0.047), and the decrement in mean arterial pressure (RM ANOVA, F = 24.2, P < 0.0001). These alterations were recovered by blood transfusions. **D** Blood withdrawal and transfusions affected shock index (RM ANOVA, F = 13.76, P = 0.007), but not perfusion index (Friedman test, P = 0.264). Data are presented as the mean ± standard error of the mean. *P < 0.05, ^†^P < 0.01 vs. baseline. BL, baseline; BW10, 10 mL/kg of blood withdrawal; BW20, 20 mL/kg of blood withdrawal; BW30, 30 mL/kg of blood withdrawal; BT10, 10 mL/kg of blood transfusion; BT20, 20 mL/kg of blood transfusion; BT30, 30 mL/kg of blood transfusion; tcPCO_2_, transcutaneous partial pressure of carbon dioxide; CO_PATD_, cardiac output measured by pulmonary artery thermodilution; CO_TPTD_, cardiac output measured by transpulmonary thermodilution; SV_PATD_, stroke volume measured by pulmonary artery thermodilution; SV_TPTD_, stroke volume measured by transpulmonary thermodilution; MAP, mean arterial pressure; HR, heart rate.

**Fig. 3:**
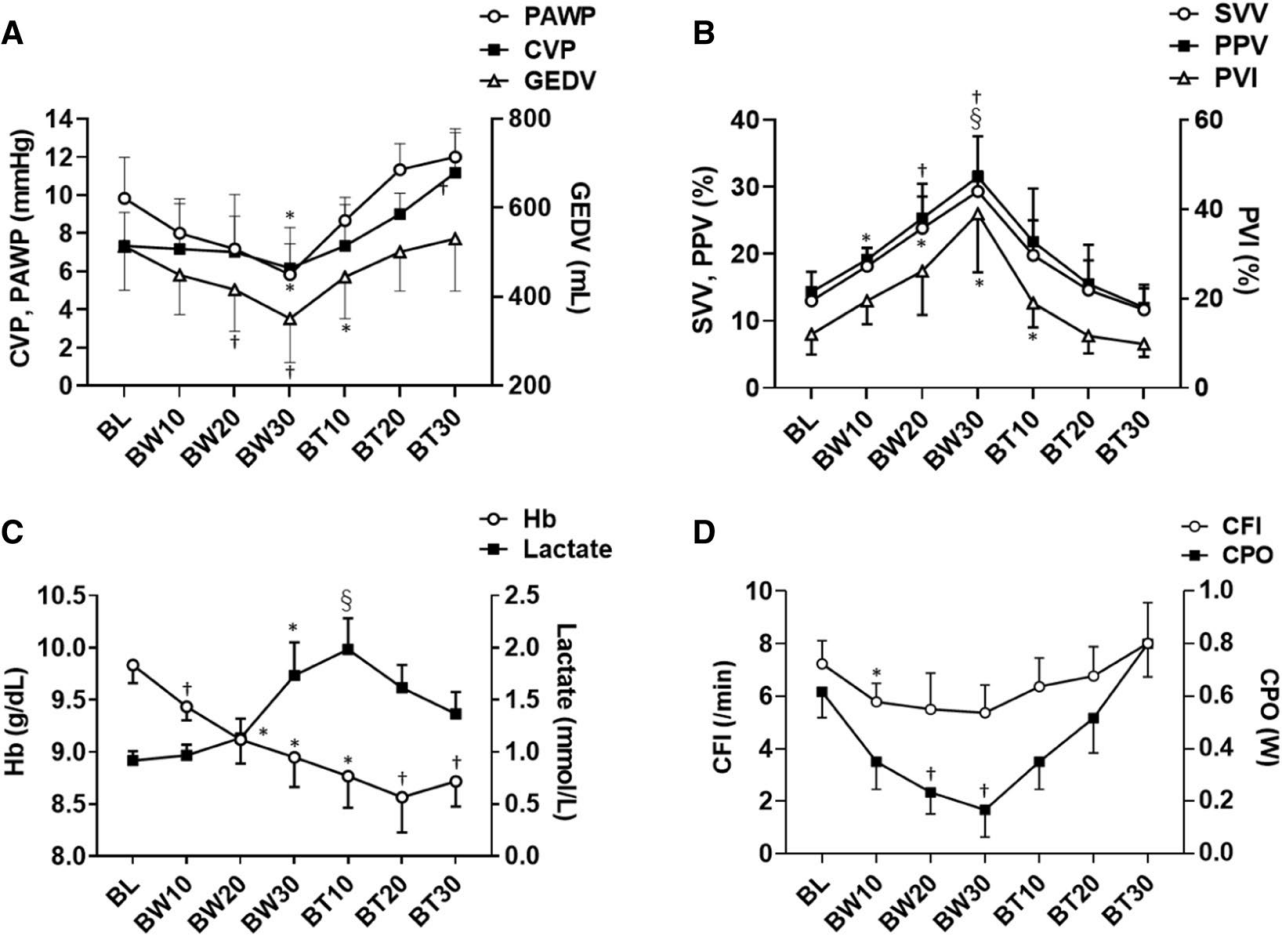
Changes in volume and perfusion parameters and laboratory data during hemorrhage shock and resuscitation. **A** Blood withdrawn induced the decrements in central venous pressure (Friedman test, P = 0.011), pulmonary artery wedge pressure (RM ANOVA, F = 18.41, P = 0.001), and global end-diastolic blood volume (RM ANOVA, F = 22.79, P = 0.0002). These alterations were recovered by blood transfusions. **B** Blood withdrawn induced the increments in stroke volume variation (Freidman test, P = 0.0001), pulse pressure variation (RM ANOVA, F = 21.01, P = 0.0002), and pleth variability index (RM ANOVA, F = 13.23, P = 0.003). These alterations were recovered by blood transfusions. **C** Blood withdrawal and transfusions affected hemoglobin (RM ANOVA, F = 12.85, P = 0.0003) and lactate levels (Freidman test, P < 0.0001). **D** Blood withdrawal and transfusions affected cardiac function index (RM ANOVA, F = 7.13, P = 0.011) and cardiac power output (Freidman test, P < 0.0001). Data are presented as the mean ± standard error of the mean. *P < 0.05, †P < 0.01, $P < 0.001 vs. baseline. BL, baseline; BW10, 10 mL/kg of blood withdrawal; BW20, 20 mL/kg of blood withdrawal; BW30, 30 mL/kg of blood withdrawal; BT10, 10 mL/kg of blood transfusion; BT20, 20 mL/kg of blood transfusion; BT30, 30 mL/kg of blood transfusion

Hemorrhage and blood transfusions impacted tc-artPCO_2_, MAP, heart rate, CVP, SI, PI, and comprehensive hemodynamic parameters obtained by PAC or PiCCO, such as CO and SV (Fig. 2). tc-artPCO_2_ increased with increased volume of blood withdrawn, whereas tc-artPCO_2_ decreased with increased BT volume (Fig. 2A). Hemorrhage and BTs markedly induced changes in preload parameters, such as CVP, PAWP, GEDV, SVV, PPV, and pleth variability index. Blood gas analyses showed significant changes in pH, artPO_2_, HCO_3_^−^, Hb, lactate, CFI, and CPO, whereas artPCO_2_ and base excess remained unchanged during the experiments (Fig. 3 and Table 1).

**Table 1:** Arterial blood gas changes during blood withdrawal and transfusion

	Status of circulating blood volume						
	BL	BW10	BW20	BW30	BT10	BT20	BT30
pH	7.51 ± 0.05	7.54 ± 0.04	7.50 ± 0.03	7.52 ± 0.08	7.46 ± 0.05	7.45 ± 0.05	7.43 ± 0.07*
artPCO_2_, mmHg	46.3 ± 6.1	44.6 ± 4.5	44.6 ± 5.0	40.6 ± 10.6	49.0 ± 4.6	50.0 ± 5.8	55.0 ± 10.3
artPO_2_, mmHg	267.3 ± 8.3	267.3 ± 6.2	275.2 ± 30.3	268.0 ± 18.1	246.2 ± 14.3*	248.3 ± 17.1	218.3 ± 42.5^a^
HCO_3_^−^, mmol/L	36.8 ± 1.8	37.0 ± 2.0	35.1 ± 1.6	34.4 ± 2.0	35.0 ± 3.2	34.6 ± 1.9*	36.3 ± 3.2
BE, mmol/L	12.4 ± 2.1	13.0 ± 1.9	11 ± 1.0	10.7 ± 2.9	10.1 ± 3.5	9.6 ± 2.4	10.7 ± 3.3

BL, Baseline; BW10, 10 mL/kg of blood withdrawal; BW20, 20 mL/kg of blood withdrawal; BW30, 30 mL/kg of blood withdrawal; BT10, 10 mL/kg of blood transfusion; BT20, 20 mL/kg of blood transfusion; BT30, 30 mL/kg of blood transfusion; pH, arterial pH; artPCO_2_, partial pressure of arterial carbon dioxide; artPO_2_, partial pressure of arterial oxygen; HCO_3_^−^, arterial bicarbonate; BE, arterial base excess

^*^P < 0.05, ^a^P < 0.01 vs. baseline

### Assessment of the agreement between tcPCO_2_ and artPCO_2_

**Fig. 4:**
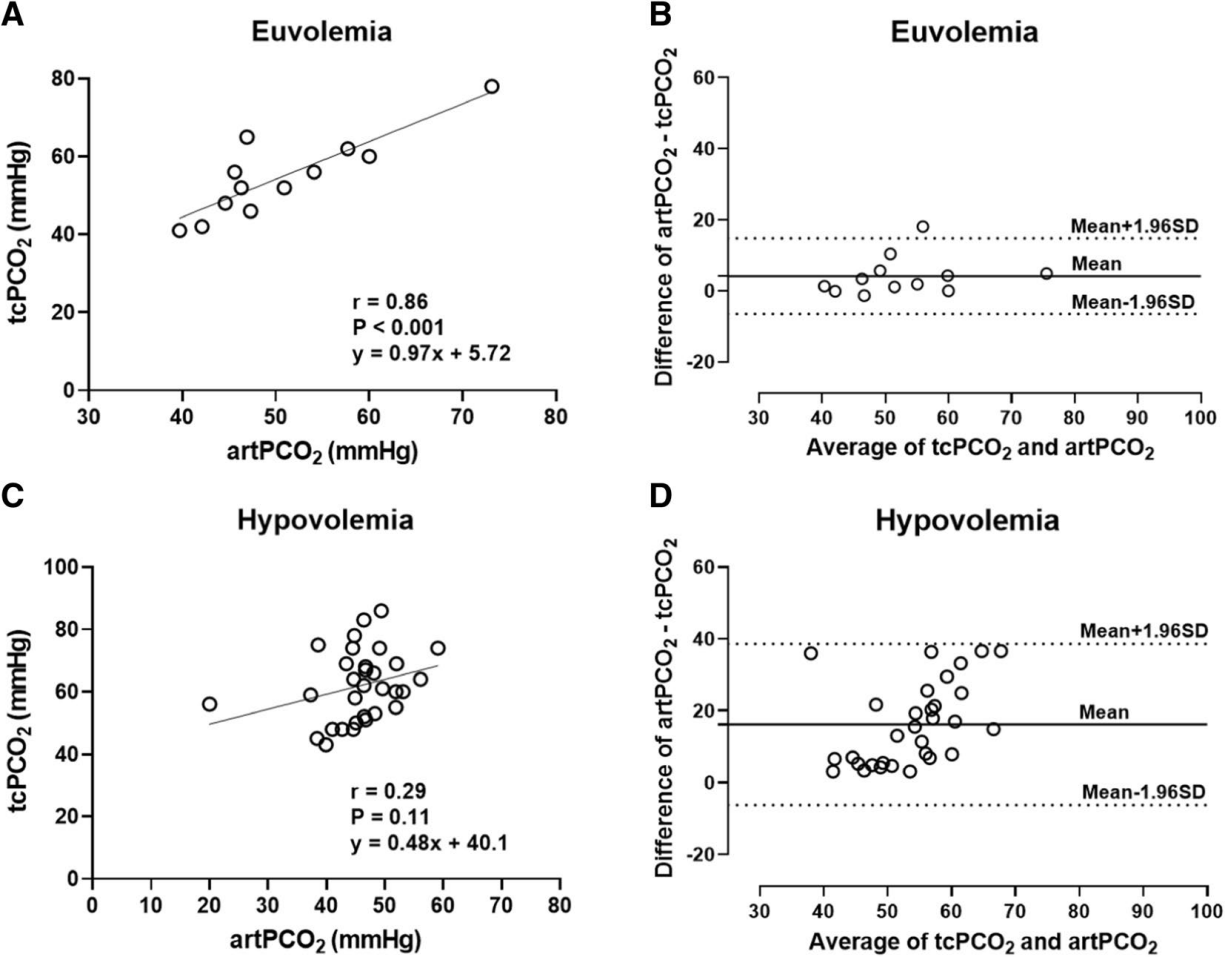
Assessments of the agreement between tcPCO_2_ and artPCO_2_ in euvolemic and hypovolemic status. **A** In the euvolemic status, a linear regression demonstrated a strong relationship between tcPCO_2_ and artPCO_2_ (r = 0.86, P < 0.001). N = 12. **B** A Bland–Altman analysis showed the mean bias was 4.1 ± 5.4 mmHg. Solid line indicated the mean difference (bias), and dash lines represent limits of agreement (mean ± 1.96 SD). N = 12. **C** In the hypovolemic status, there was no relationship between tcPCO_2_ and artPCO_2_ (r = 0.29, P = 0.11). n = 30. **D** The mean bias by a Bland–Altman analysis was 16.2 ± 11.4 mmHg. N = 30. tcPCO_2_, transcutaneous partial pressure of carbon dioxide; artPCO_2_, arterial partial pressure of carbon dioxide

To confirm that tcPCO_2_ and artPCO_2_ are coupled in normal conditions but decoupled during hemorrhagic shock, the agreements between tcPCO_2_ and artPCO_2_ were evaluated in euvolemic or hypovolemic status. Using 12 measurement points of the euvolemic status (i.e., at BL and BT30), linear regression demonstrated that there was a strong relationship between tcPCO_2_ and artPCO_2_ (r = 0.86, P = 0.0004). In Bland–Altman analysis, the mean bias was 4.1 ± 5.4 mmHg. In contrast, using 30 measurement points of the hypovolemic status, there was no relationship between tcPCO_2_ and artPCO_2_ (r = 0.29, P = 0.11), and the mean bias based on Bland–Altman analysis was 16.2 ± 11.4 mmHg (Fig. 4).

### Associations of CO with tc-artPCO_2_ and tcPO_2_

**Fig. 5:**
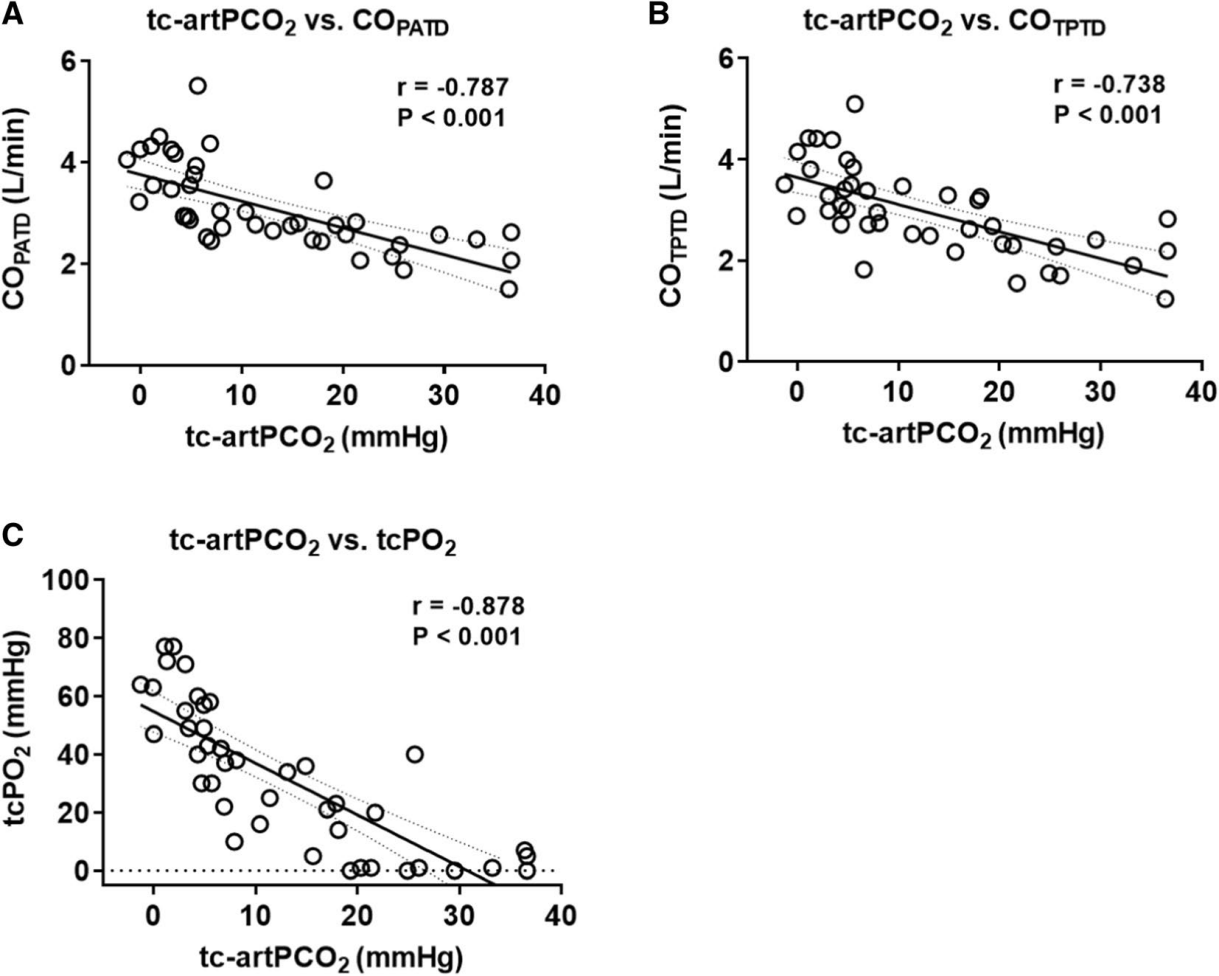
Linear regression analyses of the gradient between tcPCO_2_ and artPCO_2_ with hemodynamic parameters. There were strong relationships of tc-artPCO_2_ with (**A**) cardiac output measured by pulmonary artery thermodilution (r = −0.787, P < 0.001), **B** cardiac output measured by transpulmonary thermodilution (r = −0.738, P < 0.001), and **C** tcPO_2_ (r = −0.878, P < 0.001). Solid line represents linear regression between tc-artPCO_2_ with the parameters. Dash lines indicate 95% confidence intervals. tcPCO_2_, transcutaneous partial pressure of carbon dioxide; CO_PATD_, cardiac output measured by pulmonary artery thermodilution; CO_TPTD_, cardiac output measured by transpulmonary thermodilution; tcPO_2_, transcutaneous partial pressure of oxygen

**Fig. 6:**
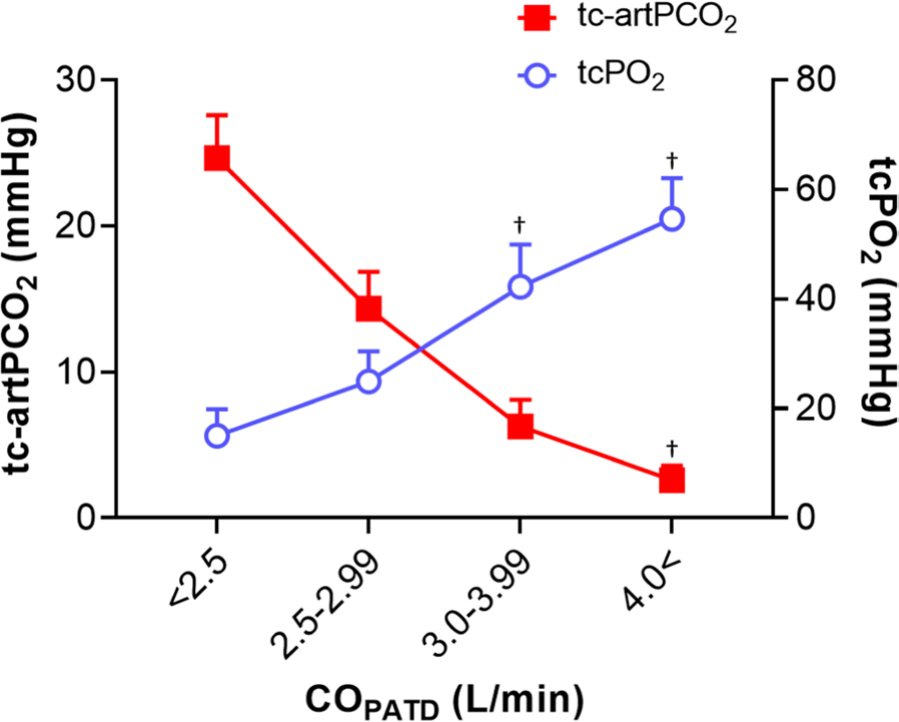
tc-artPCO_2_ and tcPO_2_ levels by cardiac output. Data are presented as the mean ± standard error of the mean. ^†^P < 0.01 vs. baseline. tc-artPCO_2_, gradient between transcutaneous and arterial partial pressures of carbon dioxide; tcPO_2_, transcutaneous partial pressure of oxygen; CO_PATD_, cardiac output measured by pulmonary artery thermodilution

Linear regressions demonstrated significant negative correlations of tc-artPCO_2_ with CO_PATD_ (r =  − 0.79), CO_TPTD_ (r =  − 0.74), and tcPO_2_ (r =  − 0.89) (all P < 0.0001) (Fig. 5). Data for tc-artPCO_2_ and tcPO_2_ obtained at all measurement time points were divided into four groups according to CO categories: CO_PATD_ (L/min) < 2.5, 2.5 ≤ CO_PATD_ < 3.0, 3.0 ≤ CO_PATD_ < 4.0, and 4.0 ≤ CO_PATD_. There was a negative linear trend between tcPCO_2_ and CO categories: with the lowest tc-artPCO_2_ value (2.6 ± 1.0 mmHg) when CO was > 4.0 L/min and the highest (24.6 ± 2.9 mmHg) when CO was < 2.5 L/min. Conversely, there was a positive linear trend between tcPO_2_ and CO categories (Fig. 6).

### Correlations between DO_2_ and hemodynamic variables

**Fig. 7:**
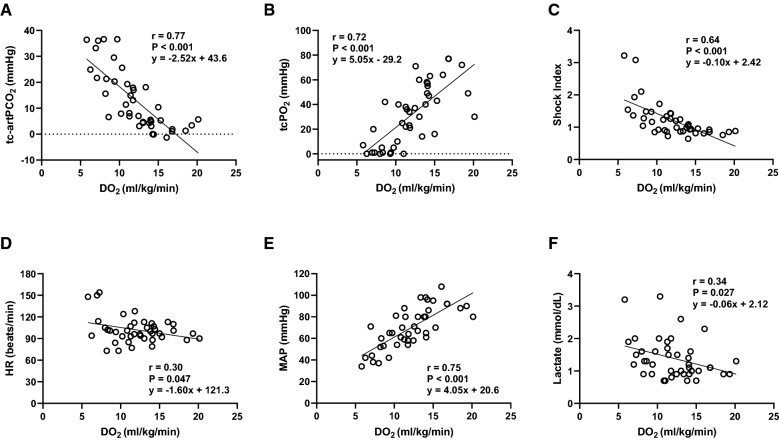
Linear regression analyses of systemic oxygen delivery and (**A**) tc-artPCO_2_, **B** tcPO_2_, **C** shock index, **D** heart rate (HR), **E** mean arterial pressure, and **F** lactate level. Liner regression analyses showed that there were strong negative correlations of systemic oxygen delivery with tc-artPCO_2_ (r = 0.77, P < 0.001) and significant positive correlations of systemic oxygen delivery with tcPO_2_ (r = 0.72, P < 0.001) and mean arterial pressure (r = 0.75, P < 0.001). N = 42. DO_2_, systemic oxygen delivery; tc-artPCO_2_, gradient between transcutaneous and arterial partial pressures of carbon dioxide; tcPO_2_, transcutaneous partial pressure of oxygen; HR, heart rate; MAP, mean artery pressure

Compared with DO_2_ at baseline (15.9 ± 2.7 mL/kg/min), significant declines in DO_2_ at BW10, BW20, BW30, and BT10 were observed (11.2 ± 2.1, P = 0.002; 9.5 ± 2.1, P = 0.018; 7.7 ± 2.1, P = 0.002; and 11.2 ± 1.5 mL/kg/min, P = 0.04, respectively). After all the stored blood was transfused (BT30), DO_2_ returned to baseline value (16.2 ± 1.9 mL/kg/min). There were significant positive correlations of DO_2_ with tcPO_2_ and MAP (r = 0.72, P < 0.001; r = 0.75, P < 0.001, respectively), and significant negative correlations of DO_2_ with tc-artPCO_2_, SI, heart rate, and lactate (r = 0.77, P < 0.001; r = 0.64, P < 0.001; r = 0.30, P = 0.047; r = 0.24, P = 0.027, respectively) (Fig. 7).

### Prediction of DO_2crit_

**Fig. 8:**
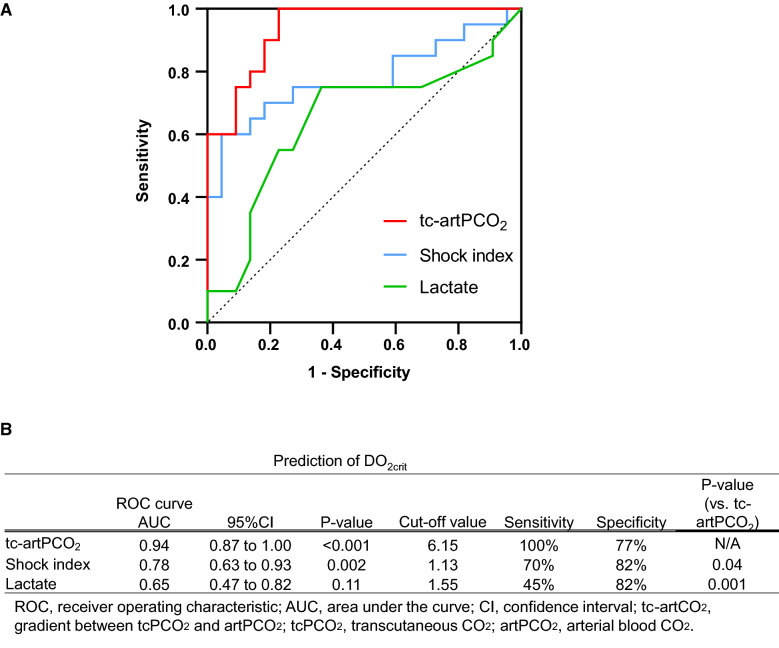
The predictive values of tc-artPCO_2_, shock index, and arterial lactate for the critical level of systemic oxygen delivery. **A** Receiver operating characteristic curves for tc-artPCO_2_, shock index, and arterial lactate to distinguish between the critical level of systemic oxygen delivery (DO_2crit_) < 11.72 and DO_2crit_ ≥ 11.72 mL/kg/min. **B** Characteristics of prognostic indexes for detecting the critical level of systemic oxygen delivery: tc-artPCO_2_, shock index, and arterial lactate

**Fig. 9:**
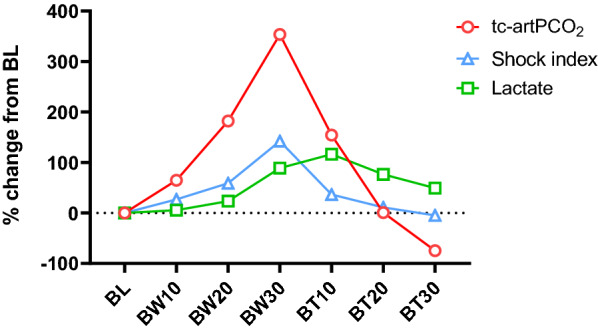
Percent changes in indicators during hemorrhagic shock and transfusions. The largest percentage change (353.3%) from the baseline was observed in tc-artPCO_2_ at 30 mg/kg of blood withdrawal. BL, baseline; BW10, 10 mL/kg of blood withdrawal; BW20, 20 mL/kg of blood withdrawal; BW30, 30 mL/kg of blood withdrawal; BT10, 10 mL/kg of blood transfusion; BT20, 20 mL/kg of blood transfusion; BT30, 30 mL/kg of blood transfusion

The critical level of DO_2_ (DO_2crit_) was calculated as 11.72 mL/kg/min, according to the predetermined tcPO_2_ threshold of 30 mmHg (see “Methods”). ROC curves were drawn to compare the predictive value of tc-artPCO_2_, SI, and lactate for DO_2crit_ (11.72 mL/kg/min). The AUCs showed that the predictive values of tc-artPCO_2_, SI, and lactate were 0.94 (95% confidence interval [CI], 0.87–1.00), 0.78 (0.63–0.93), and 0.65 (0.47–0.82), respectively, with respect to the prediction of DO_2crit_ for the transition from aerobic to anaerobic metabolism (Fig. 8A). The tc-artPCO_2_ cut-off of 6.15 mmHg provided the optimal sensitivity (100%) and specificity (77%) for predicting DO_2crit_, whereas an SI cut-off of 1.13 was identified as the optimal value (sensitivity = 70%, specificity = 82%) (Fig. 8B). The AUC for tc-artPCO_2_ was greater for predicting DO_2crit_ than for predicting SI (P = 0.04) and lactate (P = 0.001) (Fig. 8B). Figure 9 shows the percentage changes in tc-artPCO_2_, SI, and lactate at each time point, compared with those at baseline. The largest percentage change occurred in tc-artPCO_2_ with hypovolemia (BW30, 353.3%) compared with SI and lactate BW30 (142.3% and 89.0%, respectively).

## Discussion

In this study, we assessed the usefulness of tc-artPCO_2_ as a rapid yet sensitive indicator of hemorrhagic shock in a mechanically ventilated pig model during progressive hemorrhagic shock and resuscitation. We verified the hypotheses that tcPCO_2_ can non-invasively estimate and track artPCO_2_ in euvolemic conditions and that tcPCO_2_ and artPCO_2_ decoupled during hemorrhagic shock. To the best of our knowledge, this study showed, for the first time, (1) strong associations of tc-artPCO_2_ with DO_2_ and CO, which were appropriately measured based on both PAC and PiCCO, and (2) significantly superior AUC for tc-artCO_2_, compared with those for SI or arterial lactate, which are independently associated with physiological and clinical outcomes in hemorrhagic shock [1, 22, 32–34]. Taken together, we conclude that tc-artCO_2_ reflects the variations in CO and DO_2_ and can be a valid, non-invasive indicator of impaired DO_2_ during hypovolemic shock.

The primary purpose of tcPCO_2_ monitoring is to measure artPCO_2_ non-invasively and continuously, preventing the need to perform multiple blood gas analyses and guiding therapeutic interventions. In previous studies, tcPCO_2_ and artPCO_2_ reportedly decoupled during circulatory failure and are associated with poor outcomes in critically ill patients [12, 20, 21]. Additionally, tc-artPCO_2_ is less invasive, rapid, and easy to use. Therefore, monitoring tcPCO_2_ can be used as a common utility, especially in emergency departments and intensive care units. In particular, in patients on respirator, monitoring tcPCO_2_ can estimate artPCO_2_ and detect respiratory complications as a common utility, whereas tc-artPCO_2_ can estimate low CO and/or low DO_2_ for hemodynamic and metabolic assessments during shock.

Previous studies support elevated tissue PCO_2_ as a marker of hypoperfusion [35–38]. The tissue hypercarbia during shock is explained by consequent reduced removal of CO_2_ and anaerobic production of CO_2_ [39]. Current technologies allow the estimation of CO_2_ content in tissues (mucosal or cutaneous) based on the high diffusivity of CO_2_ gas. In a pig model with acute lung injury, tcPCO_2_ is an acceptable surrogate for artPCO_2_ in hemodynamically stable states; however, tcPCO_2_ and artPCO_2_ decoupled during shock [40]. This is consistent with our results. In patients with septic shock, tcPCO_2_ measured from the ear lobe at study inclusion was significantly higher in patients with septic shock than in controls. Additionally, there was a significantly greater gradient between tcPCO_2_ and artPCO_2_ or end-tidal CO_2_ (etCO_2_) in patients with septic shock than in controls [21]. Increased gastric-to-arterial PCO_2_ is an early indicator of circulatory failure and a prognostic factor of increased morbidity in patients with cardiopulmonary bypass after cardiac surgery [41]. Despite these observations, the equipment for tcPCO_2_ is not widely used in clinical practice [7] and remains poorly evaluated in preclinical large animal models.

In a pig model with lethal hemorrhagic shock, Belenkiy et al. demonstrated that the non-invasive etCO_2_ and tcPCO_2_ gradient (NICO_2_G) could serve as a proxy for the severity of hemorrhage-induced metabolic debt, as indicated by elevated arterial lactate levels [42]. Linear regression analyses of the parameters with lactate showed a low correlation for etCO_2_ (r^2^ = 0.26, P < 0.001); better for tcPCO_2_ (r^2^ = 0.46, P < 0.001); and best for NICO_2_G (r^2^ = 0.58, P < 0.0001). The findings that tcPCO_2_ increased during hemorrhage and correlated well with peripheral hypoperfusion even as artPCO_2_ remained constant [42] were consistent with our presenting results. However, etCO_2_ monitoring may not be suitable for serial hemodynamic assessments in patients with lung injury or under mechanical ventilation, as adjustments of respiratory setting could be frequently required. Additionally, etCO_2_ can be decreased in shock states because of impaired pulmonary blood flow. Thus, tc-artPCO_2_ adopted in our study might be more useful for detecting shock states in ventilated patients than NICO_2_G. Moreover, it was reported that an increase in arterial lactate occurred later than the decreases in CO and DO_2_ in a pig model with hemorrhagic shock due to a time difference in the occurrence of tissue and circulating lactic acid [43]. Thus, DO_2_ and/or CO could be more appropriate markers for shock than lactate levels. Our efforts to measure hemodynamic parameters with two different established methods (i.e., PAC and PiCCO), and using DO_2_ as a primary outcome, strengthened the results of this study.

Besides tcPCO_2_, tcPO_2_ measurement allows for continuous monitoring of tissue oxygenation, which is compromised during the early phase of hemorrhagic shock and is one of the last measures to be restored during resuscitation [44]. In this study, we further observed that tcPO_2_ responded rapidly to changes in CO during hemorrhage and blood transfusion (Fig. 6). Consistent with our results, previous studies using piglet models have demonstrated a rapid decrease in and restoration of tcPO_2_ in response to hemorrhage and fluid infusion [45, 46]. In a rat model of hemorrhagic shock and resuscitation, tcPO_2_ was more rapidly responsive than tcPCO_2_ and arterial lactate [47]. However, in clinical practice, the dependence of tcPO_2_ cannot be overemphasized [12] because tcPO_2_ depends on F_I_O_2_, which should be managed frequently during critical care. Thus, tcPO_2_ monitoring without reference to F_I_O_2_ can be misleading [29].

Our study has several limitations that should be addressed in future investigations. First, the small sample size could have resulted in a type 1 error. However, to address this limitation, we have made efforts using both PAC and PiCCO systems as hemodynamic monitors for ensuring the quality of experiments. Second, this study included only female animals to minimize heterogeneity. Future studies should include both male and female animals to investigate the role of sex in susceptibility to shock. Lastly, as this study focused only on hemorrhagic shock status, our present findings cannot be extrapolated to other shock etiologies. Thus, further studies are needed to replicate these findings for all types of shocks before extrapolating the data to other critical illnesses.

## Conclusions

Using a precise pig model, we demonstrated that continuous tc-artPCO_2_ monitoring is a minimally invasive technique that could be a rapid yet sensitive indicator of evolving hemorrhagic shock during mechanical ventilation and that tc-artPCO_2_ is superior to SI and arterial lactate levels. The present study results are encouraging, and the data clearly strengthen previous literature on techniques to monitor tissue O_2_ and CO_2_ partial pressures. Further evaluations are required in different forms of shock in other large animal models and in humans to assess its usefulness, safety, and ability to predict outcomes in critical illnesses.

## Data Availability

The datasets used and/or analyzed during this study are available from the corresponding author on reasonable request.

## Abbreviations

artPCO_2_: Arterial partial pressure of carbon dioxide
artPO_2_: Arterial partial pressure of oxygen
BL: Baseline
BT: Blood transfusion
BW: Blood withdrawal
CFI: Cardiac function index
CO: Cardiac output
CO_PATD_: Cardiac output measured by pulmonary artery thermodilution
CO_TPTD_: Cardiac output measured by transpulmonary thermodilution
CPDA-1: Citrate–phosphate-dextrose-adenine
CPO: Cardiac power output
CVP: Central venous pressure
DO_2_: Oxygen delivery
DO_2crit_: Critical DO_2_
F_I_O_2_: Fraction of inspired oxygen
GEDV: Global end-diastolic blood volume
Hb: Hemoglobin
MAP: Mean arterial pressure
PAC: Pulmonary artery catheter
PAP: Pulmonary artery pressure
PAWP: Pulmonary artery wedge pressure
PI: Perfusion index
PiCCO: Pulse index continuous cardiac output
PPV: Pulse pressure variation
PVI: Pleth variability index
SV: Stroke volume
SV_PATD_: Stroke volume measured by pulmonary artery thermodilution
SV_TPTD_: Stroke volume measured by transpulmonary thermodilution
SVV: Stroke volume variation
tc-artPCO_2_: Gradient between transcutaneous and arterial partial pressures of carbon dioxide
tcPCO_2_: Transcutaneous partial pressure of carbon dioxide
tcPO_2_: Transcutaneous partial pressure of oxygen
